# Benefits and Costs of Improved Cookstoves: Assessing the Implications of Variability in Health, Forest and Climate Impacts

**DOI:** 10.1371/journal.pone.0030338

**Published:** 2012-02-13

**Authors:** Marc A. Jeuland, Subhrendu K. Pattanayak

**Affiliations:** 1 Sanford School of Public Policy, Duke University, Durham, North Carolina, United States of America; 2 Duke Global Health Institute, Duke University, Durham, North Carolina, United States of America; 3 Nicholas School of the Environment, Duke University, Durham, North Carolina, United States of America; Universita' del Piemonte Orientale, Italy

## Abstract

Current attention to improved cook stoves (ICS) focuses on the “triple benefits” they provide, in improved health and time savings for households, in preservation of forests and associated ecosystem services, and in reducing emissions that contribute to global climate change. Despite the purported economic benefits of such technologies, however, progress in achieving large-scale adoption and use has been remarkably slow. This paper uses Monte Carlo simulation analysis to evaluate the claim that households will always reap positive and large benefits from the use of such technologies. Our analysis allows for better understanding of the variability in economic costs and benefits of ICS use in developing countries, which depend on unknown combinations of numerous uncertain parameters. The model results suggest that the private net benefits of ICS will sometimes be negative, and in many instances highly so. Moreover, carbon financing and social subsidies may help enhance incentives to adopt, but will not always be appropriate. The costs and benefits of these technologies are most affected by their relative fuel costs, time and fuel use efficiencies, the incidence and cost-of-illness of acute respiratory illness, and the cost of household cooking time. Combining these results with the fact that households often find these technologies to be inconvenient or culturally inappropriate leads us to understand why uptake has been disappointing. Given the current attention to the scale up of ICS, this analysis is timely and important for highlighting some of the challenges for global efforts to promote ICS.

## Introduction

Over half the world still uses solid biomass or coal fuels for basic cooking and heating [1]. Increasing attention is being paid to the consumption of such fuels because of their role in producing damages at three distinct scales [2]. At the household and village level, combustion of solid fuels produces pollution that is damaging to health and a large contributor to the global burden of disease [3], [4]; and imposes a high time burden on those collecting fuelwood, typically women and girls. At the community and national level, when fuel wood is harvested in unsustainable ways, its consumption contributes to the loss of forest and associated ecosystem services. Finally, at the regional and global scale, the burning of biomass and coal in inefficient household stoves, which represent roughly 15% of global energy use, releases large amounts of black carbon and carbon-based greenhouse gases [5], [6]. Many of these gases fall into the category of products of incomplete combustion, which are more damaging in terms of global warming potential than the carbon dioxide released from more fossil fuel-burning stoves [1]. These emissions contribute to global warming, particularly where such fuels are harvested non-renewably.

In fact, much of the renewed push today for improved cook stoves (ICS) stems from concerns over the contribution of traditional stoves to global climate change. This lends new impetus and a new constituency to the old idea that there are large private and social benefits from reducing reliance on inefficient biomass- or solid-fuel burning stoves [7]. In the past and today, it was and is often assumed that poor households would obviously prefer to use ICS given that traditional stoves produce large quantities of indoor air pollution. Yet progress in achieving large-scale adoption and use of ICS has been remarkably slow [8]. There is surprisingly little detailed information on uptake of ICS, but some empirical evidence suggests that high use cannot be assumed even when stoves are highly subsidized or given free of charge [9]. For example, just 45% of households in 26 villages in Peru (ranging between 6 and 71% depending on the village) used more efficient wood-burning stoves that were provided free of charge [10]. Key reasons beneficiaries cited for not using ICS are problems with stove quality, the lack of expected gains in fuel efficiency, and the difficulty or changes in cooking methods that are required for successful use. There is also evidence that individual households' propensity to use ICS may be influenced by village-level use levels (i.e. village use increases quickly as a function of household use), which echoes results on peer and network effects with other preventive health interventions [11], [12].

Meanwhile, the limited economic analyses that have been performed for ICS interventions suggest that private benefits alone should greatly exceed the costs of the stoves, throughout the developing world [7]. Given these findings, it is surprising that it has been so difficult to promote adoption and sustained use of such technologies, since households should have every incentive to invest. Yet in facing significant barriers to scaling up, ICS technologies are not unique among preventive health interventions [13], [14]. Typically, explanations for the lack of adoption focus on households' lack of understanding of health and other benefits from new technologies or on the financial barriers that preclude them making large up-front investments in such goods and services [15]. This literature does not however explain why diffusion of other technologies, such as mobile telephones, has been easier. It also glosses over questions related to preferences for ICS and the perceived and real costs of behavior change [16], including transaction and implementation costs associated with the supply-side (manufacturers, technicians, retailers, creditors) of cooking technologies.

This paper provides a simple modeling framework for systematic accounting of the costs and benefits of ICS that seeks to address many of these issues. We evaluate the move from traditional biomass-burning stove technologies to different ICS options with a simulation model, populated with uncertain parameters that (a) relate to the health, forest and climate impacts, and (b) comport to the ranges of values cited in the literature. This exercise is valuable for three main reasons. First, the elaboration of this framework and the parameterization of the model allow us to identify critical outstanding data needed to understand cook stove impacts and adoption. Second, our analysis suggests a more nuanced perspective on the economics of ICS because the costs and benefits are highly variable across typical developing country locations, and because many of the private economic benefits of ICS (e.g., time savings or reduced illnesses) are not easily perceived as tangible financial gains. The analysis focuses attention on a number of issues that may impede diffusion of ICS, including the wide variation in realized relative time and fuel use efficiencies and fuel costs, as well as in incidence and cost-of-illness of acute respiratory illness, and in the cost of household cooking time. Third, because we carry out the calculations from both a private household and social perspective, the latter of which includes forest preservation and carbon emissions reductions, the analysis provides insight on the appropriateness of carbon financing for lowering barriers to household adoption of ICS. Given the current attention to the scale up of ICS, we believe this analysis is timely and important for highlighting some of the challenges for global efforts to promote ICS.

## Methods

Our analysis compares the costs and benefits of households' switching from traditional wood-burning stoves to six alternatives: a) improved wood-burning stoves, b) unimproved charcoal-burning stoves; c) improved charcoal-burning stoves, d) kerosene stoves, e) liquefied petroleum gas (LPG), or propane stoves, and f) electric stoves. The unit of analysis for the calculations is the individual household; the monthly costs of the switch in technologies for a representative household in a developing country are compared with the monthly economic benefits that a household would receive. We compare the overall economic attractiveness of the different stove alternatives relative to the baseline of unimproved wood-burning stoves using a net benefits criterion, which is the standard economics criterion for project evaluation [17]. We also consider the transition from a traditional charcoal-burning stove to an improved charcoal-burning stove.

We conduct our assessment from both a private (household) and a social welfare perspective. For the private perspective, only the costs and benefits that accrue to households are included. In our analysis of private benefits, we also assess how capital subsidies for different stove options alter the economic benefits to households, and the extent to which varying household time preferences (based on a private rate of time preference that ranges from 10 to 20%) alters the calculation of net benefits. The social perspective accounts for the full investment and use costs of the different stoves, as well as for changes in their effects on carbon emissions and loss of forest (and uses a real social discount rate that varies from 3 to 6%). We use these calculations to motivate an analysis on how leveraging carbon finance could alter households' private net benefits for adoption and use of ICS.

### Costs and benefits associated with improved cook stoves

Each household cooking technology entails a large number of different costs and benefits. Table 1 lists the categories and includes the equations needed to compute each cost and benefit. The costs include the capital cost of a new stove and/or ventilation system, program expenses associated with distributing or marketing stoves, time and money spent for regular operation and maintenance (O&M), the net change in the cost of required fuels (which may also be a benefit depending on relative fuel costs, in time and money), and learning costs (in time and reduced quality of food preparation). Program costs include elements such as salaries and the opportunity cost of social/development workers' time, the development and logistical costs of promotion or educational campaigns, and/or any additional incentives provided to communities in order to encourage participation; these are rarely measured in a comprehensive manner in intervention studies [2], [8].

**Table 1:** Typology of costs and benefits of improved cookstoves, and equations used for calculations.

Costs	Examples	Benefits	Examples
Capital (“hardware”)[*Cap*]	Cost of new technologies: Improved cookstoves; ventilation/cooking space improvements; etc.	Morbidity & mortality reductions[*Morb*]; [*Mort*]	Benefits from reduced incidence of and mortality from disease (acute respiratory infections (esp. ALRI); COPD; etc.)
Program (“software”)[*Prog*]	Cost of implementation/delivery: Marketing and promotion materials; NGO/government staff time; etc.	Time savings[*Timesav*]	Benefits of reduced cooking time (due to more efficient heating)
Operation and maintenance[*O&M*]	Cost of replacing/cleaning of equipment, including time	Aesthetic gains	Benefits from reduced in-house exposure to unpleasant soot and smoke; reduced indoor cleaning
Fuel[*Fuel*]	Cost of fuel, in collection and preparation time and/or money	Improved social standing	Benefits of improvements in household status from acquisition of improved stoves
Learning[*Learn*]	Costs of familiarization with the use of a new stove technology	Environmental[*Carb*]; [*Bio*]	Benefits from reduced emissions of black carbon and decreased tree cutting
Inconvenience	Costs related to any undesirable changes in cooking practices made necessary by the new stove		

Notes: All parameters are defined in Table 2; unless otherwise noted here. The capital recovery factor (crf) = 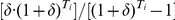, where *δ* = discount rate; and *T_i_* = lifespan of stove *i* (yrs). The following categories are not included in the model: Inconvenience costs, aesthetic gains, and improved social standing.

In the cost-benefit model, one-time capital expenses for deploying a new stove system of type *i* (*cc_i_*) are annualized using a capital recovery factor (*crf*) that is calculated based on a) the discount rate, and b) the lifetime of the stove. The capital cost incurred for the traditional baseline stove is assumed to be zero. Of course, we realize that the costs of traditional stoves cannot actually be zero; they are however orders of magnitude smaller than any of the new generation stoves available today. Annualized capital and annual program costs (*cp*) to promote the new stoves are then divided by twelve to obtain monthly capital and program costs (*Cap* and *Prog*, respectively).

In assessing O&M and fuel costs, it is important to consider only the net change of moving from traditional to improved stoves, accounting for the fact that use of the new stove may only be partial. For O&M costs this is thus the difference, weighted by a use factor χ, between monthly expenses incurred by users for the upkeep of stove *i* (*cm_i_*) and the routine operation costs of the traditional stove (*cm_0_*). For net fuel costs, the calculation of net changes is somewhat more complex. In some cases, if the cost of traditional solid fuels (including the opportunity cost of collection and fuel preparation time, if for example wood must be chopped into smaller pieces) is high compared with the cost of fuels burned in the improved stove, the net fuel costs may be negative – i.e., households realize cost savings (Note that we use the economic value of fuel collection time in this cost, to account for the fact that households may gather solid fuel rather than purchasing it in the market). This net change in fuel costs also depends on the differences in the time and quantity of fuel required for cooking [18].

In order to calculate net monthly fuel costs (*Fuel*), we start by writing the fuel consumption with the baseline stove (in kg/month):
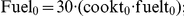
where *cookt_0_* is the average daily cooking time with traditional stove (hrs/day); and *fuelt_0_* is the quantity of fuel needed per time unit cooking for the baseline stove (kg/hr). We assume the use of fuelwood in the baseline for simplicity. Similar calculations are possible for other types of traditional fuels such as dung. The monthly cost of this baseline level of fuel consumption, in money and time, is then:
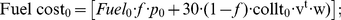
where *f* is the fraction of wood that is purchased (rather than collected); *p_0_* is the cost of the baseline fuel (in US$/kg); *collt_0_* is the average baseline daily wood collection and preparation time (hrs/day); *v^t^* is the shadow value of time; and *w* is the unskilled wage rate (US$/hr). In the above equation, the term *v^t^* is included to account for the fact that the opportunity cost of this collection time is likely lower than the unskilled wage rate.

We next derive fuel consumption for the other stoves. This is complicated by the fact that these have different fuel efficiencies (expressed in terms of useful heat provided per unit of fuel) and energy content. We write fuel consumption (in kg/month) as:
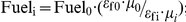
where *μ_i_* is the energy content of fuel used in stove *i* (MJ/kg fuel); and *ε_fi_* is the heat-transfer efficiency of stove *i*. These heat-transfer efficiencies are fractions that represent the amount of heat that is converted to useful cooking energy in a particular stove. Thus, the energy content of a fuel represents the theoretical amount of heat produced when burning that fuel, and the heat-transfer efficiency accounts for the amount of that heat that is transferred to food during cooking. This is multiplied by the baseline fuel amount to convert the overall expression to a fuel amount used in the improved stove.

The monthly cost of fuel consumption with the wood-burning ICS, accounting for partial use, is then:
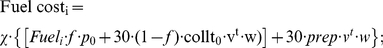
where *prep* = average time spent preparing wood for ICS stove by users (hrs/day); and all other parameters are as defined previously.

The total cost of fuel after the acquisition of the new stove is simply:
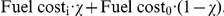



The net fuel cost (*Fuel*) of the switch to the wood-burning ICS, accounting for partial use, is thus:
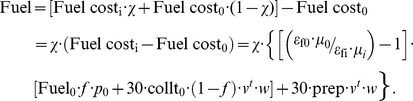



For the other stoves and fuels, we assume that the fuels are readily available and well-conditioned such that the collection and preparation time is minimal. The monthly cost of fuel consumption is thus:
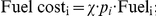
where *p_i_* is the cost of the fuel type *i* (in US$/kg, except for electric, which is US$/kW-hr). The net fuel cost (*Fuel*) for these stoves is then:
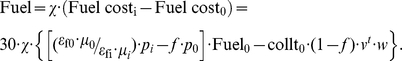



We assume that the learning cost (*Learn*) associated with improved stoves occurs shortly after the acquisition of the stove and consists of a period of self-learning of length of time *l* during which a household comes to understand how to properly use the new technology. This time is valued at the opportunity cost of time *v^t^* multiplied by the unskilled wage rate *w*. This initial learning cost is annualized and divided into monthly amounts as with the capital costs.

We calculate total costs as the sum of these components (*Cap*+*Prog*+*O&M*+*Fuel*+*Learn*). In the absence of subsidies to increase uptake of the new technologies, based on carbon-financing or other instruments, these costs will usually be privately borne and reflected in stove and fuel prices, or in time costs to the households that choose to adopt and use the new stoves. This total does not account for the inconvenience that may be associated with having to alter cooking practices to successfully use a new stove technology. We expect that such disamenities will sometimes be important to households, since improved stoves may be difficult to adapt to local cooking needs, may result in dissatisfaction with the preparation of food, or may provide less effective indoor heating during cold weather and lower protection against mosquitoes and other insects, or may not conform to individuals' preferences for cooking technology in other ways [15], [19], [20].

The benefits of ICS include health improvements from better indoor air quality, cooking time savings, aesthetic improvements and improved social standing from the use of cleaner stoves, and environmental benefits to society, such as reduced black carbon or greenhouse gas emissions and deforestation. The extent of health improvements of improved stoves are a matter of some debate. Our model only includes morbidity (*Morb*) and mortality (*Mort*) reductions due to reduced incidence of acute respiratory illness (ARI) and reduced prevalence of chronic obstructive pulmonary disease (COPD), because the evidence for reductions in other diseases, such as asthma, visual impairment, lung cancer or cardiovascular diseases, is less compelling [3], [4], [21]. For ARI, the reduction in the monthly economic cost of morbidity is estimated by multiplying the cost-of-illness (COI) per case by the decrease in expected monthly cases per household, which is a multiplicative function of disease incidence (*I^ARI^*); stove effectiveness (*η_i_^ARI^*); and household size (*hhsize*). COI includes: a) private and public expenses for diagnosis, treatment and hospitalization, b) other costs borne by patients, such as transport to hospitals, and c) productivity losses for sick patients and caretakers, during the period of illness and recovery [22]. It is important to recognize the limitations of the COI measure for measuring the economic benefits of reduced morbidity. The most important of these have to do with the fact that these benefits will be inaccurate when individuals are able to adopt behaviors that reduce their risks of illness ex ante, such that the sample of sick individuals for whom COI is known may not be representative of all affected persons. Another problem is that COI does not include the disutility associated with the non-pecuniary pain and suffering associated with an illness.

The benefits of reduced prevalence of COPD are delayed in time, because this disease results from sustained periods of exposure to poor air quality. To account for this, we multiply the decrease in prevalence of the disease, discounted by *the* number of years to disease onset (*d*), by the annual COI. We also assume that these benefits will accrue to households in direct proportion to the use rate 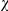, judging that most health effects studies that measure effectiveness are conducted under experimental conditions with high and sustained use. Positive externalities associated with high levels of use of improved stoves in a community are therefore not included, except insofar as they are reflected in the range of effectiveness rates reported in the literature. One might hypothesize that the relationship between health benefits and use would actually take a log or translog form, implying that approaching full use would provide diminishing marginal health benefits. If this type of relationship holds for health improvements from cook stoves, then our approach will underestimate health benefits at low use levels, and thus overestimate the effect of low use in reducing overall benefits. Alternatively, there might be thresholds below which few if any health benefits are realized, in which case our approach would overestimate health benefits for use levels below these thresholds, and thus underestimate the effect of use in reducing overall benefits, at least below the thresholds.

The reduction in the monthly economic cost of mortality is estimated by multiplying the value of a statistical life (*VSL*) by the decrease in the expected monthly risk of death per household due to the disease [23]. The VSL is typically obtained from research that studies large numbers of individuals' risk-wage tradeoffs or expenses on private goods that reduce mortality risks, for example safety products or sickness-prevention technologies such as vaccines, prophylaxis, etc. The expected risk of death is a function of the disease case-fatality rate (for ARI) or the death rate from chronic disease (for COPD). For ARI, we weight the incidence by the fraction of disease (*f*) that is acute lower respiratory illness (ALRI), based on evidence in the literature that ALRI, rather than general ARI, is the main contributor to mortality [4]. We also assume that the new fuels that are used do not have their own negative health effects, which may not be the case, particularly for kerosene [24], [25].

The nonhealth, private economic benefits of improved stoves include cooking time savings and aesthetic benefits (note that changes in collection time are accounted for above in the calculation of net fuel costs). We do not include aesthetic benefits because we are not aware of any preference studies that attempt to quantify aesthetic benefits such as the value of greater cleanliness, improved social standing, and the non-health value of reduced exposure to smoke. (Perhaps this is because it would be very difficult to obtain reliable estimates of these benefits. In theory, one could utilize carefully-designed stated preference surveys to obtain these measures, for example contingent valuation or conjoint experiments.) Monthly cooking time savings (*Timesav*) are converted to monetary benefits by multiplying the monthly time saved – obtained as the product of baseline cooking time (*cookt_0_*) and the time efficiency of stove *i* (*ε_ti_*) relative to the traditional stove – by the opportunity cost of time defined above.

Finally, we include the environmental benefits of carbon emissions savings and reduced deforestation. Because complete accounting of emissions from traditional biomass-burning stoves remains challenging and far from comprehensive, particularly with respect to the effect of black carbon [1], [5], [26], [27], we conduct analyses with two different measures of carbon intensivity for the fuel used in stove *i*. The first considers only changes in emissions of CO_2_, CH_4_, and N_2_O, as per the Clean Development Mechanism guidelines [28]. The second adds to this basic accounting the effects of CO, non-methane hydrocarbons (NMHC), and black carbon, also including for the charcoal stoves the varying intensivity of charcoal production in developing countries [29].

Following assumptions in the literature, we assume that the biomass burned in cook stoves is sustainably harvested. If the harvesting of biomass fuel is unsustainable, this will increase the carbon intensity of the fuel, since on net the emissions are not being recycled into the regrowth of forests. However, a reasonably careful review of the literature finds no rigorous study that quantitatively demonstrates if the wood used in cookstoves is harvested sustainably or not. The increasing rates of forest degradation and deforestation in many parts of the world suggest that harvesting is more likely unsustainable. Unfortunately, only Bond et al. [12] present illustrative figures suggesting that unsustainable harvesting could double 20-yr warming potential, and increase 100-yr warming potential even more, but the question remains as to what fraction of wood harvesting is actually sustainable in different locations. The net change in emissions is thus a function of the change in the amount of fuel burned in the new stove multiplied by that fuel's specific emissions intensivity, which depends on its energy content *μ_i_* (defined above) and its carbon intensivity factor *γ_i_* (in g CO_2_ equivalent/MJ useful energy). This change is valued at the cost of carbon (in US$/ton CO_2_ eq) to yield carbon savings *Carb*.

Because we assume sustainable harvesting of fuel wood, our valuation of reduced deforestation and degradation (*Bio*) is based on the replacement cost for trees (in US$/kg wood) [7]. A preferable economic value for the benefit of reduced deforestation would be a measure of the actual value of forest services that are lost due to wood harvesting – e.g., impacting water flow, soil erosion or species habitat, which could theoretically rely on estimates from the forest valuation literature [30]. The difficulty in producing such a calculation for our analysis lies in translating forest values, usually measured in $/hectare for specific eco-regions and climates, into a global measure $/kg of wood, which requires information on variation in yields in different locations among other challenges.

Total benefits are the sum of the components *Morb*+*Mort*+*Timesav*+*Carb*+*Bio*. Total private benefits include only the first three of these terms, unless some climate benefits are passed on to private households via carbon financing. There could also be indirect health benefits because of infectious disease dynamics, for example, if the incidence rates of ARI decrease at the community level due to many users of improved stoves, which in turn lowers individual exposure to the infectious ARI agents. However, we are not aware of scientific studies that demonstrate changes in disease risks at the population level as a function of uptake of improved cooking technologies, and so do not include health externalities in our analysis.

### Data and modeling approach

In order to estimate the costs and benefits of switching cookstoves, we conducted an extensive review of the literature in order to specify the values of the approximately thirty parameters that appear in the equations for costs and benefits (see Table 2). For each model parameter, Table 2 shows the range of plausible values obtained based on our reading of the literature for “typical” programs designed to promote different cookstove technologies (and also lists the studies that provide this information).

**Table 2:** Definition of model parameters for analysis of costs and benefits of cook stove technologies.

Parameter	Description	Unit	Low	Mid	High	Sources
*cc_i_*	Cost of stove type *i*	US$				[4], [7], [21], [37], [38]; www.consumerreports.org
	Improved wood-burning only (ICS)		5	15	50	
	Traditional charcoal-burning		3	4.5	6	
	Improved charcoal-burning		3	14	50	
	Kerosene		10	30	60	
	Propane (LPG)		60	90	120	
	Electric		100	300	500	
*δ_s_*	Discount rate (social)	None	3	4.5	6	Judgment
*δ_p_*	Discount rate (private)		10	15	20	
*T_i_*	Lifespan of stove *i*	yrs				[7]; www.consumerreports.org
	Improved burning only		2	3	4	
	Traditional charcoal-burning		2	3	4	
	Improved charcoal-burning		2	3	4	
	Kerosene		4	5	6	
	Propane		5	10	15	
	Electric		10	15	20	
*cp*	Cost of promotion of new stoves, assumed to be the same for all stove types	US$/hh-yr	0.2	2.0	3.8	[4], [39]
*χ*	Sustained use of new stove	%	0.2	0.5	0.8	[10], [40]–[41]
*cm_i_*	Cost of stove maintenance					[39]
	Traditional wood-burning (*i* = 0)	US$/hh-yr		0		
	All other stoves			1.4		
*cookt_0_*	Average daily cooking time with wood stove	hrs/day	2	3	4	[21], [39], [42], [43]
*εt_i_*	Time efficiency of stove *i* relative to traditional stove	Fraction of time with improved stove				[2], [7], [8], [18], [39], [35], [40], [44]
	Improved wood-burning		0.7	0.95	1.5	
	Traditional charcoal-burning		0.6	0.75	1.0	
	Improved charcoal-burning		0.6	0.75	1.0	
	Kerosene		0.5	0.7	0.9	
	Propane		0.45	0.67	0.9	
	Electric		0.35	0.63	0.9	
*p_i_*	Cost of fuel type *i*					[7], [18], [38], [45]–[49]
	Wood (*i* = 0)	$/kg	0.03	0.12	0.2	
	Charcoal	(Except electric, in $/kW-hr)	0.1	0.45	0.8	
	Kerosene		0.3	0.5	0.7	
	Propane		0.4	0.7	1.0	
	Electric		0.03	0.065	0.10	
*f*	Percentage of people buying wood	%	0	25	50	
*fuelt_0_*	Amount of fuel per hr spent cooking; traditional stove	kg/hr	0.3	0.6	1.0	[7], [18], [45]
*εf_i_*	Fuel efficiency of stove *i*	MJ useful energy/MJ produced heat, except for electric				[18], [27]
	Traditional wood-burning		7%	11%	15%	
	Improved wood-burning		13%	25%	40%	
	Traditional charcoal-burning		18%	20%	21%	
	Improved charcoal-burning		15%	26%	37%	
	Kerosene		40%	45%	50%	
	Propane		50%	55%	60%	
	Electric (kW-hr needed per hr cooking)		1.10	1.65	2.20	
*collt_0_*	Average daily wood fuel collection time	hrs/day	0.3	1.0	3.0	[7], [39], [50]
*prep*	Average daily fuel preparation time for ICS stove	hrs/day	0.17	0.33	0.50	
*hhsize*	Number of persons per household	persons/hh	4	5	6	
*I^ARI^*	Incidence of ARI	cases/person-yr	0.1	0.5	1.0	[51]–[53]
*P^COPD^*	Prevalence of COPD	%	1	4.5	8	[2], [51], [54], [55]
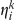	Reduction in disease *k* from use of improved stove *i*	%				[4], [21], [29], [39], [40], [56]
	Improved wood-burning only (ARI)		10	40	70	
	Improved wood-burning only (COPD)		0	15	30	
	Traditional charcoal-burningl (ARI)		0	20	40	
	Traditional charcoal-burning (COPD)		0	5	10	
	Improved charcoal-burningl (ARI)		10	40	70	
	Improved charcoal-burning (COPD)		0	15	30	
	Kerosene (ARI)		45	60	75	
	Kerosene (COPD)		0	20	40	
	Propane (ARI)		45	60	75	
	Propane (COPD)		0	20	40	
	Electric (ARI)		45	60	75	
	Electric (COPD)		0	20	40	
*COI^k^*	Cost-of-illness of disease *k*					
	ARI (nonsevere cases)	US$/case	2	15	60	[54], [57]
	COPD	US$/yr	30	35	40	Pattanayak [personal comm.]
*d*	Delay in onset of COPD symptoms	yrs	10	15	20	
*VSL*	Value of a statistical life	US$/life lost	10000	30000	50000	[58], [59]
*f^ALRI^*	Fraction of all ARI that is severe ALRI	None	0.04	0.15	0.25	[3], [60]
*CFR^ALRI^*	Case fatality rate of ALRI	lives lost/case	0.01	0.03	0.05	[51]–[53], [57], [61], [62]
*drate^COPD^*	Mortality rate due to COPD	deaths/10,000	0	1	2	[51]
*v^t^*	Shadow value of time spent cooking (fraction of market wage)	None	0.1	0.3	0.5	Judgment and value of time studies [63]
*w*	Unskilled market wage	US$/hr	0.13	0.2	0.5	
*ccarb*	Cost of carbon emissions	US$/ton	5	20	35	
*μ_i_*	Energy conversion factor for stove *i*					[27], [47]
	Wood	MJ/kg fuel (except electric MJ/kW-hr)		16		
	Charcoal			30		
	Kerosene			35		
	Propane			45		
	Electric			3.6		
*γ_i_*	Carbon intensity of fuel^1^					
	Wood	g CO_2_ eq per MJ (Except electric, in g/kW-hr)		12.1		[1], [5], [27]
	Charcoal			5.6		EIA: http://www.eia.doe.gov/oiaf/1605/coefficients.html
	Kerosene			157.4		
	Propane			107.9		
	Electric (varies by source of power)		70	170	270	
*ce*	Cost of tree replacement	US$/kg	0.002	0.01	0.02	[7]

1 Only includes CO_2_, N_2_O and CH_4_. For the sensitivity analysis with accounting for CO, NMHC and black carbon, we adjust the overall emissions values from Figure 6 of Bond et al. [12] based on the mid-level efficiency *εf_i_* of the typical stoves to obtain the following emissions intensities, all in g CO_2_ equivalent/MJ: Wood = 225; Charcoal = 410; all others same as above.

As shown, the parameters quoted in the literature vary greatly, and some have scarcely been documented, for example the relative time spent preparing fuel for use in different stoves. Therefore, methods that have been used for calculating costs and benefits in past analyses of improved cook stoves, and that use average parameter values, create a risk that the economics of stove alternatives will be misinterpreted. To better characterize the uncertainty in outcomes, we adapt the simulation approach developed by Whittington et al. [22] – first used for comparing improvements in water and sanitation – to determine the net benefits of households' switch from traditional stoves to the alternative stoves. Specifically, we conduct two types of analyses: a) Monte Carlo simulations of the net benefits for the various stove options, allowing all uncertain parameters to vary simultaneously; and b) one-way parameter sensitivity tests, presented as tornado diagrams, which generate insights concerning the factors most important in affecting economic outcomes [31].

In our Monte Carlo simulations, all cost-benefit model parameters are assumed to be uniformly distributed over the specified ranges because we have no data on the true statistical distributions of these model parameters across developing country locations. Our analysis thus aims to uncover the extent of possible outcomes given reasonable parameter values, drawn from the literature, for a range of such locations. In addition, we specify likely correlations, also included in the online appendix, between parameters in the model in order to avoid putting undue emphasis on what we consider to be particularly unlikely combinations of model parameters (Table 3).

**Table 3:** Assumed model parameter correlations^1^.

Parameter	Symbol	Correlated parameters	Justification
Cost of stove *i*	*cc_i_*	Lifespan of stove *i* (0.7)Wage rate (0.5)Time efficiency of stove (−0.5)Fuel needed per unit time (0.5)Reduction in both diseases (0.5)Case fatality rate/death rate (0.5)	More durable stoves may cost moreStove costs may be higher in richer placesMore efficient stoves may cost moreMore efficient stoves may cost moreCleaner stoves may cost moreStove costs may be higher in isolated places with poor health care
Lifespan of stove *i*	*T_i_*	O&M cost (0.5)Baseline cooking time (−0.5)Time efficiency (−0.5)	Better O&M may lengthen stove lifeMore time cooking may reduce stove life
Program cost	*cp*	Incidence, prevalence (0.5)Case fatality rate/death rate (0.5)Wage rate (0.5)	Program costs may be higher in isolated places with poor health careProgram costs may be higher in richer places
O&M cost	*cm_i_*	Baseline cooking time (0.5)Wage rate (0.5)	More time cooking may increase O&M needO&M cost includes time spent cleaning
Baseline cooking time	*cookt_0_*	Wood fuel needed per unit time (−0.5)Shadow value of time (−0.5)	People may reduce cook time if opportunity cost and fuel requirement is higher.
Cost of wood/charcoal fuel	*cf_i_*	Wage rate (0.5)	Fuel costs may be higher in richer places
Shadow value of time	*μ*	Cost of wood/charcoal fuel (0.5)Wage rate (0.5)	The relative value of time gathering fuel may be lower if market prices for fuel are highThe value of time gathering fuel may be higher where wage rate is higher
Incidence of ARI	*I^ARI^*	Wage rate (−0.5)	Incidence of ARI may be higher in poor places with low wages
Cost of illness of disease *k*	*COI^k^*	Wage rate (0.5)	Cost-of-illness includes lost productivity
Value of a statistical life	*VSL*	Wage rate (0.7)	VSL depends on income
Case fatality rate from ALRI	*CFR^ALRI^*	Wage rate (−0.5)	Case fatality rate from ALRI may be higher in poor places with low wages

1 Correlations only listed once.

A probabilistic sensitivity analysis is then conducted, in which net benefits are calculated for each of the improved stove options for 10,000 realizations of values for the parameters in the model. This yields a distribution of net benefit outcomes for each of the stoves, which is associated with the ranges of parameter values that we think are likely to exist in developing countries. Since these ranges have been informed by published information in the literature, we would expect to find site-specific circumstances in developing countries with a similar range of outcomes. We emphasize that the frequency with which any specific combination of parameter values – or net benefit outcomes – would arise is unknown. As a result, these cumulative distributions should not be interpreted to represent the precise distribution of outcomes.

As far as we know, ours is the first attempt to characterize the extent of uncertainty in outcomes based on real data from developing country locations. Hutton et al. [7] use WHO region averages for their cost-benefit calculations. Mehta and Shahpar [4] do the same from a cost-effectiveness perspective. Our calculations also differ from these previous studies because we take the household as the unit of analysis, rather than calculating outcomes for entire regions. We believe this to be a useful perspective because successful achievement of large-scale dissemination of non-traditional stove technologies ultimately must rely on households changing behavior and their costs-benefits calculus. Understanding the variation in private benefits from these stove alternatives is a critical first step in thinking about what factors are likely most critical to increasing or suppressing consumer demand for households. Similarly, the relationship between private and social benefits can provide insight on public interventions, and especially whether carbon finance based subsidies to households can tip the scales toward adoption.

## Results

We present four sets of results of our cost-benefit modeling of the switch to improved stove technologies that use the same fuels (wood- or charcoal-burning), or to stoves that use different fuels (kerosene, LPG and electric). First, we present results from a private household perspective, without subsidies. We then present calculations that include social costs and benefits, using the UNFCC accounting methodology that focuses on CO_2_, CH_4_, and N_2_O emissions, and assessing the potential for carbon financing to enhance private demand. Third, we show the main drivers of variations in these private and social net benefit outcomes. Finally, we consider the effect of including more types of stove emissions (CO, NMHC and black carbon) that are thought to contribute to global warming.

### Household private net benefits from different stove options

**Figure 1:**
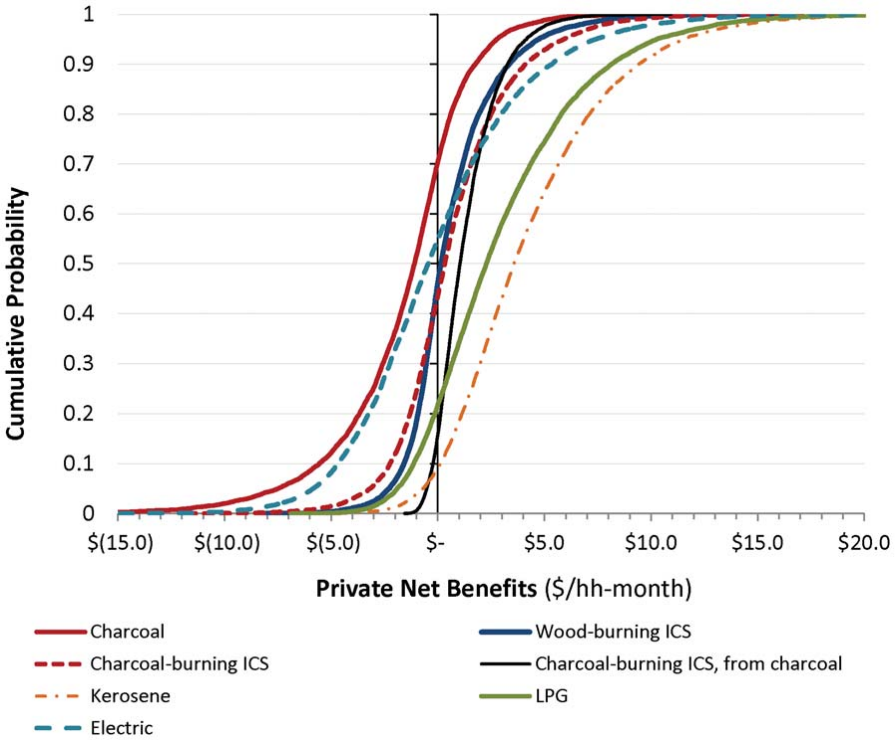
Private net benefits of different stove options. All are measured relative to traditional wood-burning stoves except for the move from the traditional to improved charcoal-burning stove.

The simulations of different stove technologies show that variability in the parameters that determine costs and benefits translates into a wide spread of potential economic outcomes. From a private perspective, the most generally attractive decision would involve switching from traditional wood-burning stoves to kerosene or LPG, or from traditional to improved charcoal-burning stoves (Figure 1). Still, a large majority of simulations for these three changes in cooking technologies result in welfare gains (positive net benefits), and many combinations of parameters for LPG and kerosene appear particularly beneficial in terms of the magnitude of net benefits. For the switch from traditional wood-burning stoves to improved wood-burning, improved charcoal-burning, or electric stoves, just about half of the simulations result in positive net benefits; the switch from traditional wood-burning to unimproved charcoal-burning stoves appears least beneficial. The electric stoves and unimproved charcoal-burning stoves both yield some outcomes with strongly negative net benefits.

### Social net benefits of different stove options, with basic carbon emissions accounting

**Figure 2:**
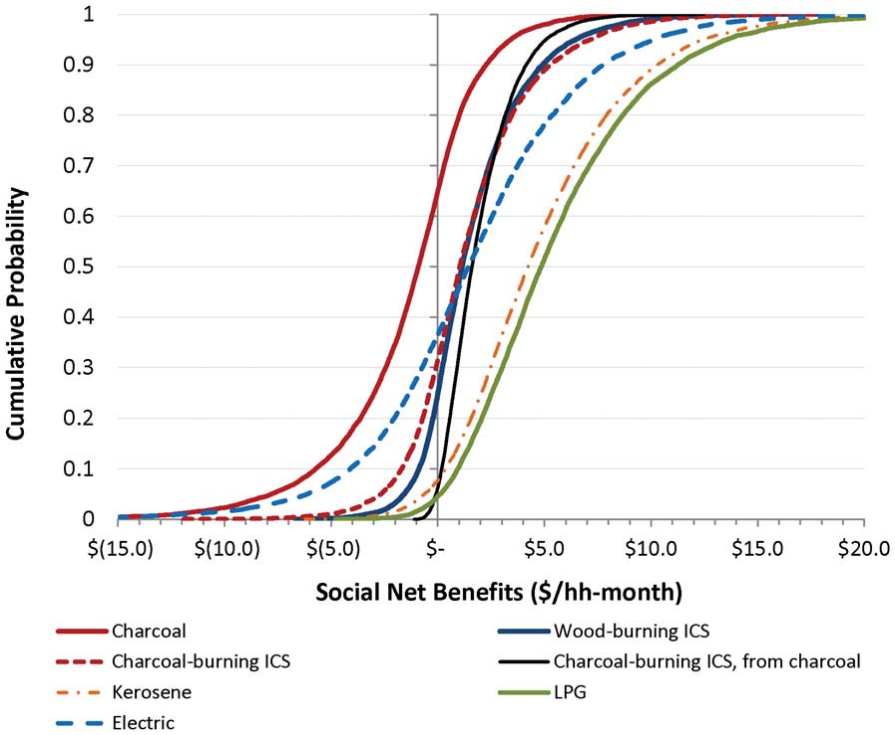
Social net benefits of different stove options, with UNFCC methodology accounting for emissions from CO_2_, CH_4_, and N_2_O. All are measured relative to traditional wood-burning stoves except for the move from the traditional to improved charcoal-burning stove.

Since avoided carbon emissions and other social benefits (reduced forest loss) provide much of the rationale for renewed focus on subsidies to incentivize cookstove adoption, we now turn to the analysis of the social net benefits of these different options. We begin by considering the results that include only carbon emissions from CO_2_, CH_4_ and N_2_O, which are those most commonly included in such calculations [28]. Similarly to the analysis that only considers private benefits, inclusion of these additional categories of social benefits does not always result in positive net benefits (Figure 2). It is true that the economics of some stoves – notably the LPG and wood-burning ICS stoves – are modestly better when this basic carbon accounting is done. This is because these cleaner-burning and efficient stoves lead to somewhat reduced emissions overall. Such stoves would therefore seem to be good candidates for carbon financing or carbon offset subsidies, but the subsidies would perhaps only have modest impacts on household uptake of the improved stoves. Other stoves do not look much better with inclusion of these benefits (for example kerosene and unimproved charcoal). This is because burning such fuels is actually not much cleaner than use of wood fuels in traditional stoves, at least in terms of CO_2_, CH_4_ and N_2_O emissions.

### Parameters that drive variation in private and social net benefits, and emissions outcomes

**Figure 3:**
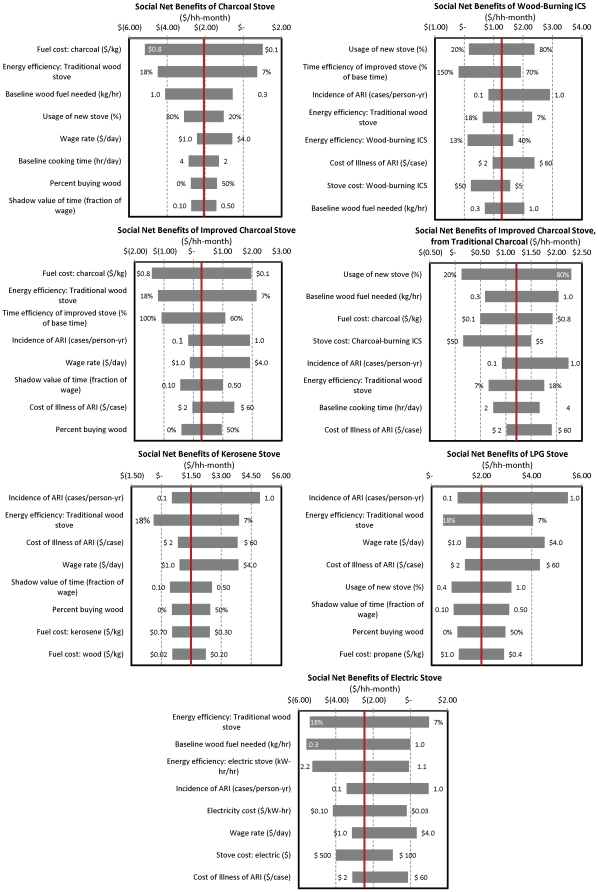
Parameters that drive changes in social net benefits of different stove options, with UNFCC methodology accounting for emissions from CO_2_, CH_4_, and N_2_O. All measured relative to traditional wood-burning stoves except for the move from the traditional to improved charcoal-burning stove (Panel D). The red line shows the outcome for the midpoint parameter values.

To better understand these results, we next consider the parameters that drive the variation in private and social net benefits, using one-way sensitivity analyses for the different stove alternatives. Figure 3 shows that the contribution of different parameters to overall social benefits varies by stove. Consider for example the wood-burning ICS (Panel B). The most important factors influencing the net benefits of the switch to this stove are the use of the stove and its relative time efficiency (compared to the traditional stove). These parameters are important because a large proportion of the benefits of this stove come from time savings, but these are only captured if it is used often and efficiently. Inefficient stove use imposes a net time cost on users. With all other parameters held constant, we see that the net benefits from low (20%) to high (80%) use increase from being barely positive ($0.10/hh-month) to about $2.40/hh-month. Other important parameters for this stove are the incidence of ARI and the cost of illness of ARI, which determine health benefits, and the relative energy efficiencies of the traditional versus improved stoves. For the charcoal stoves, on the other hand, the most important drivers tend to be in parameters that affect the relative cost of fuel: the market price of charcoal, and the amount of baseline fuel needed and baseline energy efficiency, which influence the relative gains obtained from the new stove. Also important are the use rates, the market wage and baseline cooking time, the latter two of which determine the value of collection and cooking time savings (Panels A, C and D). For the kerosene and propane stoves, the incidence and cost-of-illness of ARI, which determine some of the health gains, figure much more prominently. Also important are the value of time savings (determined by relative time efficiency, market wage, and shadow value of time savings) (Panels E and F). Finally, net benefits of the electric stove are most strongly affected by its relative efficiency and the stove and electricity prices (Panel G).

The factors that matter for private net benefits are only slightly different from those that matter for social net benefits (Results not shown; tornado charts for private net benefits and the value of emissions savings similar to those presented in Figure 3 are available upon request). Stove and fuel costs become more critical factors, particularly for the wood-burning and charcoal-burning ICS, and the electric stoves (stove cost), and the charcoal stoves (fuel cost). The parameters related to time efficiency parameters and to acute respiratory illness (which determine private time and health savings) play a more important role in overall private net benefits, while the parameters related to energy efficiency and fuel use (which determine the social benefits from decreased emissions) play a reduced role. Interestingly, neither the private rate of time preference nor any of the stove life parameters figure prominently in changing the simulated outcomes. The discount rate is only the ninth most significant parameter in shifting outcomes for the electric stove, and is less important for the other technologies.

**Figure 4:**
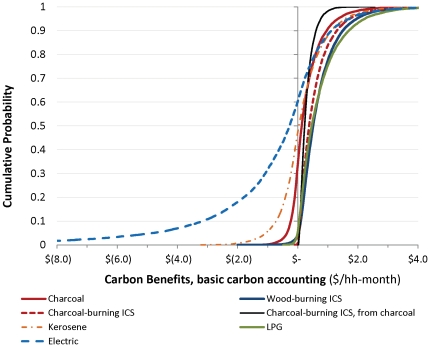
Carbon emissions benefits for different stove options, with UNFCC methodology accounting for emissions from CO_2_, CH_4_, and N_2_O. All are measured relative to traditional wood-burning stoves except for the move from the traditional to improved charcoal-burning stove.

To better dissect the changes in our results for carbon emissions, and to motivate the question of the role of carbon finance or carbon subsidies, we first look at the ranges of the carbon benefits from the stoves alone (Figure 4). The net carbon benefits for the LPG and wood- or charcoal-burning ICS are almost always positive, using the most basic carbon accounting for these options. This is consistent with the previously observed rightward movement of the distribution of social net benefits relative to private benefits for these options. For the electric or kerosene stoves, on the other hand, 50% or more of the simulated outcomes yield net emissions cost for reasons described below. The net change in emissions cost is also ambiguous for the unimproved charcoal stove.

The factors driving the spread in simulated emissions benefits vary depending on the stove. For the wood-burning ICS and the various charcoal-burning stoves, this spread is primarily determined by the energy efficiency of the traditional stoves (lower efficiency improves savings), the cost of carbon emissions (higher values imply greater benefits), and stove use rates (higher use of more efficient stoves increases savings). The spread in the value of changes in emissions from the LPG stove is driven by similar factors, as well as by the amount of fuel used in the baseline stove (higher amounts lead to greater savings). For the kerosene and the electric stoves, the sign of carbon savings depends almost completely on the relative energy efficiencies of the traditional stove and the new stove; higher relative kerosene or electric stove efficiency leads to savings, otherwise these imply net costs. The emissions intensity of the electricity generation process supplying the electric stove is also critical.

### Effect of different emissions accounting on household net benefits and implications for carbon finance

It is often claimed that one of the major barriers to adoption of some of the more advanced stoves and fuels (e.g. LPG and electric) is the investment finance needed to support household adoption of new stoves. As a result, carbon finance or “Pigouvian” subsidy is seen to be the key strategy for facilitating household adoption – i.e., households deserve payments (subsidies) for providing global public goods. In this section, we summarize the low (10^th^ percentile), median and high (90^th^ percentile) indicators from our distributions of private net benefits, first without subsidy, and then adjusted to fully include a subsidy or tax that internalizes the basic emissions outcomes for greenhouse gases included in the standard carbon emissions accounting methodology [28]. In other words, we add the net value of carbon benefits, calculated in each simulation, to the private benefits for each stove option presented previously. We then test the sensitivity of these results to the more complete accounting of emissions as presented in Bond et al. [5].

As shown in Table 4, the transfer of emissions offset subsidies to households based on a basic accounting methodology would improve outcomes from a private perspective for several stoves, most notably the wood- and charcoal-burning ICS, and the LPG stoves. All three indicators (low, median and high) for these stoves improve when the carbon offsets are subsidized. For the electric stove, the low and median outcomes actually get worse (moving from a loss of $4.7 to $6.6/hh-month at the 10^th^ percentile, and from a loss of $0.5 to $0.9/hh-month at the median) because electricity generation often relies on coal or emissions-intensive processes. With kerosene, outcomes at the left of the distribution of net benefits become worse (the 10^th^ percentile net benefits move from being marginally positive at $0.1 to negative −$0.1/hh-month). Taken together, these results suggest that carbon subsidies designed on the basis of this simple accounting could help improve the economics of stoves to some degree, thereby increasing the incentives to take up some improved stoves in many locations. However, this would be socially sub-optimal in 10% of the cases – i.e., combinations of poorly performing stove, low use rates, and limited health benefits.

**Table 4:** Ranges of private net benefits of different stove options (relative to traditional wood-burning stoves, except for charcoal as indicated) as a function of the amount of capital subsidy, and ranges of overall social benefits (All in $/hh-month; parentheses indicate negative outcomes).^1^

Stove option	Private benefits:No stove subsidy			Social benefits:Basic carbon accounting^2^			Private benefits with carbon offset subsidy: Basic carbon accounting^2^			Private benefits with carbon offset subsidy: Additional emissions accounting^2^		
	Low	Median	High	Low	Median	High	Low	Median	High	Low	Median	High
Charcoal	($5.6)	($1.1)	$1.8	($5.7)	($0.9)	$2.3	($5.5)	($0.9)	$2.2	($8.1)	$1.7	$18.1
Improved wood stove	($1.6)	$0.2	$3.3	($0.9)	$1.1	$4.9	($1.2)	$0.8	$4.4	$1.5	$10.0	$29.3
Improved charcoal	($2.2)	$0.3	$4.1	($1.7)	$1.0	$5.3	($1.8)	$0.8	$5.0	$0.7	$7.9	$26.4
Improved charcoal, from basic charcoal	($0.2)	$1.0	$3.3	$0.2	$1.6	$4.1	($0.1)	$1.3	$3.8	$1.6	$5.5	$13.4
Kerosene	$0.1	$3.6	$9.4	$0.3	$4.2	$10.3	($0.1)	$3.8	$9.8	$9.9	$23.8	$51.0
Propane	($1.1)	$2.3	$8.1	$0.9	$4.9	$11.2	($0.7)	$3.0	$9.2	$8.9	$22.9	$50.7
Electric	($4.7)	($0.4)	$5.4	($4.1)	$1.4	$7.8	($6.6)	($0.9)	$5.3	$4.0	$18.4	$46.9

1 Low and high correspond to the 10^th^ and 90^th^ percentile outcomes from the simulations.

2 Basic carbon accounting includes CO2, N2O and CH4, as specified in the UNFCC guidelines (UNFCC 2010), whereas additional accounting adds CO, NMHC and black carbon, following Bond et al. [5].

**Figure 5:**
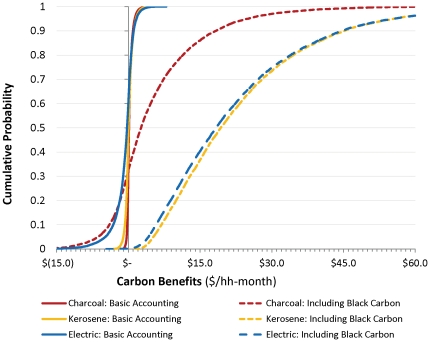
The effect of using different accounting assumptions about emissions from unimproved charcoal, kerosene and electric stove (measured relative to traditional wood-burning stoves). Basic accounting includes only CO_2_, CH_4_ and N_2_O; the other also includes CO, NMHC and black carbon.

These results change dramatically if the emissions accounting includes CO, NMHC, and most importantly, black carbon. We caution that the more complete emissions estimates come from a study which synthesizes results from various published papers but nonetheless admits them to be preliminary [5]. Still, given the calculations of strongly positive net benefits (rightmost columns of Table 4), even much more modest assumptions about emissions savings should be sufficient to justify offset subsidies and promote adoption. Even the 10^th^ percentile net benefits for the modern stoves (kerosene, LPG and electric) are now strongly positive (ranging from about $4 to $10/hh-month), and both wood- and charcoal-burning improved stoves would also deliver benefits at the left-hand side of the distribution ($0.75–1.60/hh-month), though these would be lower because black carbon and other emissions would not be eliminated. Median and 90^th^ percentile outcomes are very large with this more complete accounting. In some cases, at the left side of the distribution, unimproved charcoal stoves remain worse than traditional wood stoves, for example if their efficiency is low or the charcoal-production process is inefficient. Figure 5 makes clearer how this different accounting affects the calculation of carbon benefits for the three stoves with the poor outcomes under the basic accounting method (unimproved charcoal, kerosene, and electric).

## Discussion

This paper considered the private and social implications of household use of cooking technologies in developing countries. Working from a typology of the different categories of benefits and costs developed to describe such changes, a model was created for simulating net benefits. This model was structured to reflect some of the realities and challenges associated with shifts in cooking technologies, by allowing for the possibility of both gains and losses in time and fuel savings, as well as various levels of use, as in Whittington et al. [13]. The model was then parameterized using values from the scant but growing empirical literature on improved cook stoves and cooking fuel use in the developing world.

Such a model structure is consistent with field evidence on the heterogeneity of user experiences and real world efficiencies of these types of technologies. For example, it is often claimed that ICS interventions will result in time and fuel savings for households. Yet many interventions that focus on dissemination of purportedly ‘efficient’ technologies have suffered because the ways in which users change behaviors lead to no change or net increases in time spent cooking or preparing fuels [32]–[35]. Some evidence from the field even points to increased firewood consumption in ICS (Nepal et al. 2011). In such cases, health benefits will often be reduced, and use of the “improved” technology is likely to decline further. Similarly, some types of stoves may be ill-suited for particularly important cooking tasks; for example, Masera and Omar [36] cite the difficulty of using LPG stoves to prepare tortillas and other “traditional foods” in Mexico. For many stoves, user behavior – improper fuel loading, lack of maintenance – appears to result in efficiency losses. To avoid such problems and properly instruct households on proper use, producers of improved stoves need to understand heterogeneous consumer preferences and provide customer support and technical assistance.

Our results go part way to showing the importance that these factors may play in adoption and long term use of improved cook stoves. We confirm conventional economic claims that time efficiency and the opportunity cost of time are critical factors in affecting the relative private returns of improved cooking technologies compared to traditional stoves using solid fuels. To be sure, health benefits will in many cases be important for households, but these are dependent on use, which is ultimately conditional on perceptions of the convenience and adaptability of the improved stoves to their cooking tasks. Because health benefits are non-pecuniary or at best averted expenses (e.g., reduced costs of illness), they may not be as salient to households as the ordinary daily costs of fuel purchase or time spent using stoves.

Changes in relative fuel and stove costs have the largest influence on net benefits from the charcoal-burning and more expensive LPG and electric stove options. Policy-makers seeking to foster greater adoption of cleaner stoves might therefore target these items through price subsidies, perhaps on the basis of calculations similar to those presented in this paper. However, there are several potential problems with such a strategy. First, there are many technical questions about the accounting of emissions. We demonstrated that these assumptions can lead to dramatically different conclusions about the appropriateness of carbon finance. Currently, there remains considerable debate in the scientific community about how to do this accounting correctly. We need improved methodologies for dealing with black carbon and the extreme heterogeneity in emissions from different types of stoves, both inherent in design and related to the way individuals use them.

Second, we showed that the capital cost of the stoves plays a relatively modest role in determining private net benefits, which are much more dependent on use rates, time savings, and relative fuel costs. As a result, free or high-subsidized distribution of new stoves, even if justified from a carbon accounting perspective, may not lead to widespread use if that use is itself costly. Third, fuel subsidies for non-solid fuels may be subject to capture by wealthier households who already own those improved stoves, or may actually encourage over-use of fuels, thereby increasing pollution. Thus, it is hard to imagine that strategies which subsidize either stoves or fuel would succeed; both would probably be necessary, and perverse incentives that increase fuel use (and decrease stove efficiency and therefore carbon benefits) would probably be inevitable.

Although the framework presented in this paper is useful for comparing different stove types, there are very clear limitations to the analysis. First, the ranges for the parameters used in the model have been informed by published information in the literature, and likely represent the variety of site-specific circumstances in developing countries, but the joint distributions of those parameters are unknown. For this reason, the frequency with which any specific combination of parameter values – and resulting net benefit outcomes – would arise in the real world is unknown. Because of this uncertainty about frequency distributions of parameters and outcomes, the relative rankings and or net benefits of different stoves shown by the cumulative probability distributions in this paper may be somewhat inaccurate. For example, too much weight may have been ascribed to parameters that have a disproportionately negative impact on certain stove types. Still, we do not believe that such shortcomings would alter the conclusion that ICS interventions may generate a wide range of economic outcomes.

Second, there are limitations related to the model construction of costs and benefits. For one, many parameters in the model were specified based on the results of two or three intervention studies in different parts of the developing world. Generalization from such a small set of rigorous studies is risky, and it is thus reasonable to expect that the ranges of variation in outcomes may be even larger than is shown here. More specifically, program costs for scaled-up cook stove interventions are entirely unknown at this time; research is required to better understand these. This should be troubling considering the extent of investment that is likely to occur in this domain in the near future due to growing concerns over climate change, facilitated by the United Nations' Global Alliance for Clean Cookstoves (GACC; more information available at: http://cleancookstoves.org/overview/). The effect of black carbon, and the shortcomings in the accounting system being used in the Clean Development Mechanism more generally, also requires a great deal more study. We include the value of reduced deforestation in a very simplistic way, assuming that such resources are harvested sustainably, and therefore utilizing the concept of replacement cost for lost biomass. Where non-renewable harvesting is the norm, the impetus for subsidizing a shift towards more efficient cooking technologies will be greater.

Furthermore, aesthetic benefits and disamenities related to different cooking options have not been included. These and health benefits may in fact be highly concentrated on certain members of the households, such as the women who do much of the household cooking in developing countries, or young children who tend to stay closer to their mothers and the kitchen. Understanding the implications of this for household decision-making (e.g., whether female-headed households make different choices than male-headed households) requires careful and more extensive survey work that feeds into a more general theory of adoption and use, of which there is surprisingly little, particularly with regards to cooking technologies [9], [16].

Finally, and perhaps most importantly, we are not providing explanations of how households will behave in the real world. Indeed, the model developed in this research is not an attempt to explain the adoption problem because it is parameterized with real site-specific evidence rather than data that reflects the full range of (perhaps incorrect) household perceptions of impacts. Instead, what it offers is a description of simulated economic outcomes related to likely household behaviors, based on information on experiences pertaining to cook stove interventions in developing countries. We find this description – which suggests considerable heterogeneity in outcomes for all stoves – to be plausible and critical for identifying the key challenges to renewed attempts to scale up cook stove interventions.
